# The Clinical Significance of Pancreatic Steatosis in Pancreatic Cancer: A Hospital-Based Study

**DOI:** 10.3390/diagnostics14192128

**Published:** 2024-09-25

**Authors:** Chia-Hao Chan, Chia-Chen Chang, Yen-Chun Peng

**Affiliations:** 1 Department of Radiology, Taipei Veterans General Hospital Taitung Branch, Taitung 950410, Taiwan; lohengrinchan@hotmail.com; 2 Department of Radiology, Taichung Veterans General Hospital, Taichung 407219, Taiwan; 3 Department of Medical Imaging, China Medical University Hospital, China Medical University, Taichung 404327, Taiwan; flpalm51@gmail.com; 4 Division of Gastroenterology, Department of Internal Medicine, Taichung Veterans General Hospital, Taichung 407219, Taiwan; 5 School of Medicine, National Yang Ming Chiao Tung University, Taipei 112304, Taiwan

**Keywords:** pancreatic steatosis, fatty pancreas, fatty infiltration, pancreatic cancer

## Abstract

**Background/Objectives:** Pancreatic cancer remains one of the deadliest malignancies worldwide with a pressing need for early detection and intervention strategies. Emerging evidence has suggested a potential link between pancreas steatosis, characterized by excessive pancreatic fat accumulation, and an increased risk of pancreatic cancer development. This retrospective imaging study aims to elucidate the association between pancreatic steatosis and the subsequent development of pancreatic cancer. In the study, we aimed to determine the characteristics of pancreatic cancer with pancreatic steatosis. **Methods:** During the period of January 2022 to December 2022, we conducted a retrospective study, collecting 101 newly diagnosed pancreas cancer cases from the available image datasets. A comprehensive database of retrospective abdominal imaging studies, comprising computed tomography (CT) and magnetic resonance imaging (MRI), was established from a diverse patient population and subsequently analyzed. Inclusion criteria encompassed patients having available baseline imaging data, allowing for the assessment of pancreatic fat content. Pancreatic fat content was quantified using validated radiological techniques, while demographic, clinical, and histopathological data were all collected. The clinical data and patient characteristics were collected from medical records and analyzed. **Results:** Preliminary analysis revealed a significant correlation between elevated pancreatic fat content and an increased incidence of subsequent pancreatic cancer. Moreover, subgroup analysis based on age, gender, and comorbidities provided valuable insight into potential risk factors associated with this progression. Additionally, the study identified novel radiological markers that may serve as early indicators of pancreatic cancer development in individuals with pancreatic steatosis. **Conclusions:** In the imaging study, approximately 30% (30/101) of pancreatic cancer patients presented with pancreatic steatosis. Chronic pancreatitis emerged as the primary factor contributing to pancreatic steatosis in these patients. Importantly, pancreatic steatosis did not significantly impact the prognosis of pancreatic cancer. Follow-up data revealed no significant differences in survival duration between patients with or without pancreatic steatosis. Additionally, no association was found between pancreatic steatosis and hepatic steatosis.

## 1. Introduction

Pancreatic cancer is a less common cancer, with a growing trend in incidence worldwide. Additionally, the incidence of pancreas ductal adenocarcinoma is also increasing [1]. Concurrently, chronic pancreatitis is considered to carry the risk of developing pancreas cancer [2]. There are a wide range of causes for chronic pancreatitis, including alcoholism, hyperlipidemia, autoimmunity, trauma, and idiopathic disease, as well as other reasons [3]. Research also highlights the core genetic mutations and pathways involved in pancreatic cancer, providing insights into the molecular mechanisms driving the disease [4]. There is a growing concern that these metabolic changes can have both a physiological and pathological impact on human health. While metabolic steatohepatitis remains a risk for hepatic carcinogenesis [5], a less focused pancreatic disease, pancreatic steatosis, should also be paid attention to because of its association with pancreatic carcinogenesis.

The causes of pancreatic steatosis have not yet been well investigated; therefore, its association with chronic pancreatitis has also not been well defined [6]. Pancreatic steatosis, also known as fatty pancreas, is a condition characterized by an abnormal accumulation of fat within the pancreatic tissue [7,8]. The incidence of pancreatic steatosis occurs in approximately 16% of people found in the global population [6]. Pancreatic steatosis has been studied using CT scans in 1995 and can be diagnosed through the images created by several modalities [7,9,10]. The diagnosis and assessment of pancreatic steatosis are typically conducted through imaging modalities such as computed tomography (CT) or magnetic resonance imaging (MRI) [11], both of which are non-invasive techniques that provide detailed images of the pancreas [7,12]. However, the clinical significance of pancreatic steatosis has also been understudied [7,13,14], with its association with pancreas cancer requiring further investigations [15,16].

The presence of uneven fatty deposits within the pancreas has been documented in previous studies, with the deposits predominantly concentrated in the anterior aspect of the pancreatic head [8,9,17]. The relationship between pancreatic function and pancreatic steatosis has been explored, revealing that the impact of pancreatic steatosis on endocrine function outweighs its effect on exocrine function [8,18]. It is worth considering pancreatic steatosis in the context of non-alcoholic steatohepatitis, as it is associated with various metabolic functions. However, the precise connection between pancreatic steatosis and metabolic syndrome remains unclear [19]. Previous investigations into pancreatic steatosis and its potential link to pancreatic malignancy have highlighted male gender as a significant factor. Notably, this study emphasizes the utility of sonography as a primary diagnostic tool for identifying pancreatic steatosis [20]. There is increasing evidence that pancreatic steatosis is associated with pancreatic cancer, as seen in both animal and human studies [15,21]. There has been a study that showed weight loss associated with pancreas fat accumulation [22]. A recent study also disclosed that gut microbiota with pancreas steatosis focuses on gut microbiota and pancreas steatosis [23]. The issue of the connection between pancreatic steatosis and pancreatic cancer should be more of a concern to the medical community.

The etiology of pancreatic steatosis and its connection with pancreatic malignancy remains an area which requires both further exploration and clarification [24]. Despite a recent analysis showing an association between pancreatic steatosis and pancreatic cancers, the included studies seemed to be limited, and the cause of pancreas steatosis seemed to not be clarified [25]. The relationship between pancreatic steatosis and pancreatic cancer has not yet been extensively studied. In this current study, our objective is to investigate the association between pancreatic steatosis and pancreatic cancer. Through this investigation, we aim to identify potential indicators or risk factors that may shed light on the connection between pancreatic steatosis and pancreatic cancer as we conduct a retrospective analysis involving a series of consecutive cases of pancreatic cancer in our hospital. Through a thorough review of imaging data, we aimed to evaluate the presence of pancreatic steatosis. Additionally, we examined clinical data extracted from medical records in order to assess the clinical significance of pancreatic steatosis in the context of pancreatic malignancy.

## 2. Materials and Methods

### 2.1. Subjects

This retrospective study on pancreatic cancer, which had been diagnosed using CT or MRI, involved reviewing radiological images taken between January 2022 and December 2022 and comparing the interpretations of at least two radiologists. To conduct this study, the initial step was to compile a comprehensive dataset of patients who underwent imaging studies during the specified time frame. This dataset included pertinent patient information such as age, gender, medical history, and presenting symptoms. Once the dataset was established, the CT or MRI scans of the identified patients were collected and organized. These scans served as the primary source of data for the study and provided valuable imaging findings or staging such as tumor size and location, associated lesions, and the presence of metastasis. In order to gain a complete understanding of the patient’s condition, other relevant clinical information such as lab test results or histopathology reports was included. This study was approved by the Institution Review Board of Taichung Veterans General Hospital (CE24054B). The diagnosis of chronic pancreatitis is established based on medical history, risk factors, underlying predispositions, and clinical presentations, including the presence of chronic abdominal pain, a history of recurrent episodes of acute pancreatitis, and symptoms indicative of pancreatic exocrine insufficiency, such as diarrhea, steatorrhea, or unexplained weight loss. The diagnostic confirmation is further supported by cross-sectional imaging modalities, such as CT or MRI, which may reveal structural changes consistent with chronic pancreatitis [26].

### 2.2. Pancreatic Steatosis

**Figure 1:**
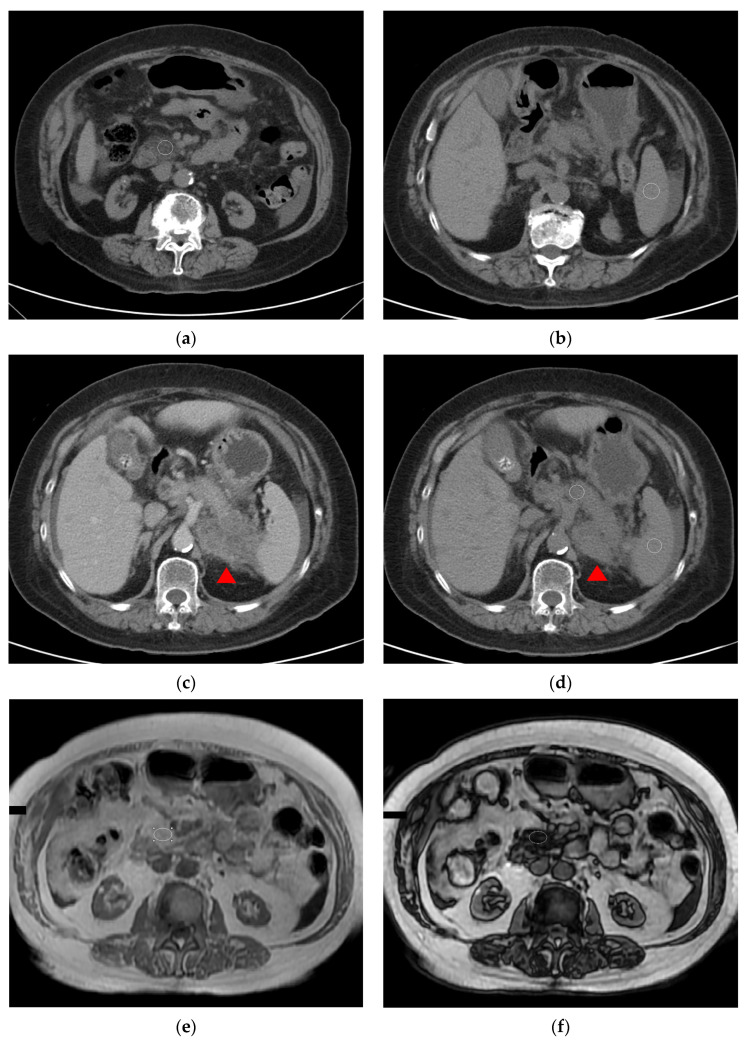
(**a**) Example of a patient with pancreas cancer and pancreatic steatosis in a non-contrast-enhanced CT scan (the main tumor site was located at the pancreas tail and was not shown), with measurement at the pancreas about average at −14 HU in the circular area. (**b**) Example of a patient with pancreas cancer and pancreatic steatosis in a non-contrast-enhanced CT scan (the main tumor site was located at the pancreas tail and was not shown), with measurement at the spleen about average at 44 HU in the circular area. (**c**) This image showed pancreatic cancer in the pancreatic tail (red triangle) on the contrast-enhanced CT scan. (**d**) This image showed the same level on the non-contrast-enhanced CT scan compared with Figure 1c. The circular area on the pancreas avoids the tumor site (red triangle) and reveals an average HU of about 23, and the circular area on the spleen reveals an average HU of about 44. (**e**) Example of a patient with pancreas cancer and pancreatic steatosis in an in-phase MRI (the main tumor site was located at the pancreas tail and was not shown), with the measurement at the pancreas about average at 859 in the circular area. (**f**) Example of a patient with pancreas cancer and pancreatic steatosis in an out-phase MRI (the main tumor site was located at the pancreas tail and was not shown), with measurement at the pancreas about average at 153 in the circular area.

Subsequently, the interpretations of pancreas cancer and pancreatic steatosis made by at least two radiologists were reviewed and compared. This step is essential in order to identify any discrepancies or variations in the diagnoses provided. Consistency in identifying pancreatic cancer and any differences in tumor staging or characterization were evaluated in the radiologists’ reports. In CT scans, according to the previous study, images at the non-contrast-enhanced phase should be acquired for actual attenuation, with comparisons between the pancreas and spleen or liver then suggested. In this study, the comparison between the pancreas and spleen was conducted because excessive fat deposits in the liver (fatty liver) is a common disease that may interfere with the results, while the spleen’s attenuation remains relatively consistent. The diagnosis of a fatty pancreas was confirmed when the measuring area’s average attenuation for the pancreas was below the spleen. (Figure 1a,b). The measuring area avoided the tumor site with contrast-enhanced CT scan (Figure 1c) and non-contrast-enhanced CT scan (Figure 1d) due to every enrolled patient having been diagnosed with pancreatic cancer, as well as due to other obvious pathologic findings such as a cyst.

Through the use of an MRI scan, the application of the in-phase and out-of-phase sequences is to identify the pathological (microscopic) fat content of tissues by showing a drop in signal intensity on the out-of-phase sequence images as compared to the in-phase images. The Chemical Shift Imaging or Dixon method employs both in-phase and out-of-phase sequences to distinguish fat from water-containing tissues. Regarding out-of-phase images, the signal from fat cells that infiltrate the pancreas will cancel out the signal from the surrounding water-rich pancreatic tissue, leading to areas of signal loss that represent the fatty infiltration. However, the previous study suggested that MRI proton density fat fraction (MRI-PDFF), proton magnetic resonance spectroscopy (1H-MRS), or iterative decomposition of echo asymmetry of water and fat with least squares estimation (IDEAL) were all golden or accurate noninvasive quantitative assessments for pancreatic fat accumulation. However, these sequences were not performed in our routine sequence during abdominal surveys. The chosen measuring area was similar to a CT scan but remained the same size and site between in-phase and out-of-phase in order to obtain accurate results. (Figure 1e,f).

Both a CT scan and MRI can also help assess the pancreas for any concurrent pathological conditions associated with fatty infiltration, such as pancreatitis or tumors. It is important to recognize that while the presence of fat in the pancreas can be benign, particularly in individuals who are overweight or have metabolic syndrome, its presence may also be associated with chronic pancreatic diseases and potentially contribute to pancreatic inflammation and fibrosis. Therefore, the detection and monitoring of pancreatic steatosis through the use of a CT scan or MRI remain crucial for the clinical management of patients both for diagnostic and prognostic purposes.

Overall, a retrospective study on pancreatic cancer diagnosed by either a CT scan or MRI involves collecting and organizing imaging and clinical data at a specific time frame. The interpretations of at least two radiologists are compared, and their agreements or disagreements are then examined. Conducting such a study enables researchers to gain insight into the accuracy and consistency of pancreatic cancer diagnosis using these imaging modalities, thus identifying areas for improvement, and opening avenues for further research.

### 2.3. Statistical Analysis

Continuous variables, which include measurable quantities that can vary along an interval, are scrutinized using a range of methods that both describe and infer relationships. Descriptive statistics such as the mean and median summarize the central location of the data, while measures like standard deviation and variance illustrate spread. When hypotheses regarding population means are tested, inferential statistics come into play. The *t*-test, for instance, compares the means of two groups. For categorical variables, which represent data sorted into distinct categories, the analysis shifts to frequency distributions. The chi-square test assesses the independence between two categorical variables.

Regression analysis is pivotal for continuous variables. But since further analysis was not performed on certain patients due to there being only one variable, chronic pancreatitis was significant.

In our Kaplan–Meier analyses, patients were censored when they no longer provided additional information for the analysis. When the last clinical fact in the patient’s record fell within the time window for analysis, they were censored on the day following that last fact in their record.

## 3. Results

### 3.1. Characteristics in Pancreas Cancer Patients with and without Pancreatic Steatosis

Amongst the 101 patients with pancreas cancer diagnosed using either a CT scan or MR, the data and images were reviewed by two doctors through their medical records. The characteristics of the patient groups are shown in Table 1. Overall, there were 30 pancreas cancer patients with pancreatic steatosis and 71 patients without. Thus, the percentage of pancreatic steatosis was seen in approximately 30% of pancreas malignancies in the present study. The basic data were analyzed and revealed no differences in age, gender, BMI, pathology, or whether the patient had received surgery.

### 3.2. Chronic Pancreatitis Is Associated with Pancreatic Cancers with Pancreatic Steatosis

Chronic pancreatitis seemed to be the most significant factor between pancreas cancer patients with pancreatic steatosis or without (13/71, 18.3% vs. 13/30, 43.3%, *p* < 0.009). Thus, chronic pancreatitis was the most significant factor for pancreatic steatosis in pancreas cancer patients.

### 3.3. Factors Associated with Mortality in Pancreas Cancer Patients

We conducted a comprehensive analysis of clinical factors, including pancreatic steatosis, in patients diagnosed with pancreatic cancer. Our initial analysis identified several key factors associated with patient mortality. (Table 2) Specifically, we found that body mass index (BMI), the decision to undergo surgery, and the staging of pancreatic cancer were significantly associated with mortality outcomes in these patients. Conversely, pancreatic steatosis and chronic pancreatitis did not demonstrate clinical significance in relation to patient survival. To further elucidate these associations, we performed a multivariate analysis using Cox regression. (Table 3). This advanced analysis reaffirmed the initial findings, highlighting that the decision to undergo surgery and a diagnosis of stage IV pancreatic cancer were strongly associated with increased mortality risk. These results underscore the critical impact of surgical intervention and cancer staging on the prognosis of pancreatic cancer patients while also indicating that pancreatic steatosis and chronic pancreatitis may not be significant prognostic factors in this patient population.

### 3.4. Survival Analysis

**Figure 2:**
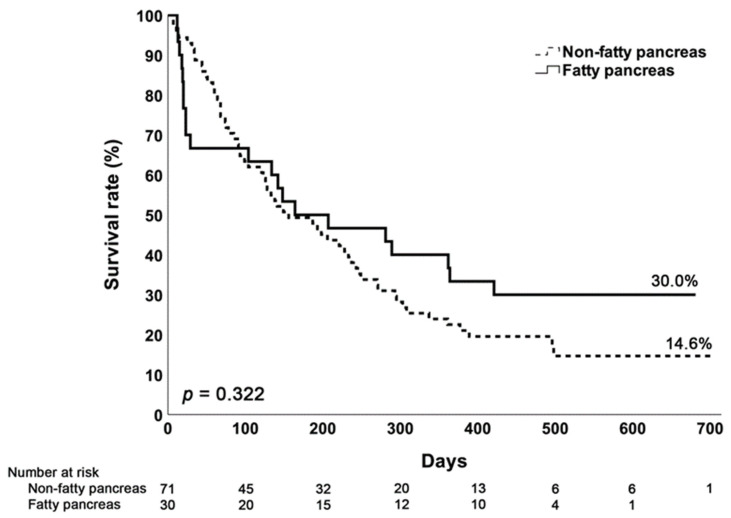
The Kaplan–Meier curve in pancreas cancer patients with and without a fatty pancreas. The results show that the percentage of survival seems to be higher in patients with a fatty pancreas, but the difference is not significant (*p* = 0.322).

All patients included in this study were monitored with regard to their follow-up and survival statuses. The follow-up period was concluded on either 31 December 2023 or upon the date of death. This period was calculated from the index date of patients included in the study starting from 1 January 2022. Survival curves were analyzed using the Kaplan–Meier method. Our findings revealed no significant difference in the average follow-up or survival duration between pancreatic cancer patients with or without pancreatic steatosis (Figure 2).

### 3.5. Association of Pancreatic Steatosis and Hepatic Steatosis

Hepatic steatosis is now recognized as being associated with various metabolic disorders. To this aim, we conducted an analysis to examine the association between these two conditions. Table 4 uses the chi-square test for statistical analysis, and our results indicated that there was no significant association between the presence of pancreatic steatosis and hepatic steatosis. The chi-square test did not reveal a statistically significant relationship, suggesting that the occurrence of steatosis in the pancreas does not necessarily correlate with the occurrence of steatosis in the liver.

## 4. Discussion

Approximately 30% (30/101) of pancreatic cancer patients were found to have pancreatic steatosis. Our study demonstrated that chronic pancreatitis is the primary factor contributing to pancreatic steatosis in these patients. There were no significant differences in age, gender, or surgical interventions among those diagnosed with pancreatic cancer. Pancreatic steatosis did not significantly impact the prognosis of pancreatic cancer. Mortality analysis highlighted the critical role of surgical intervention and cancer staging in prognosis, whereas pancreatic steatosis and chronic pancreatitis were not significant prognostic factors. Further follow-up showed no significant differences in follow-up time or survival duration between patients with or without pancreatic steatosis. Additionally, no association was found between pancreatic steatosis and hepatic steatosis in this study.

Pancreatic cancer is a multifactorial disease with complex processes and several risk factors contributing to its development and progression. Chronic pancreatitis has been consistently linked to an increased risk of pancreatic cancer due to prolonged inflammation and subsequent genetic mutations [26,27]. Diabetes mellitus, particularly type 2, has also been associated with pancreatic cancer [28]. Smoking is also considered one of the risk factors, with tobacco carcinogens inducing direct damage to pancreatic tissue [29]. Genetic predisposition plays a significant role, with mutations in genes such as KRAS, CDKN2A, and BRCA2 being prevalent among familial cases [30]. Obesity and high BMI contribute to the risk of pancreas carcinogenesis, likely due to metabolic disturbances and chronic persistent inflammation [31]. Understanding these factors is crucial for early detection and developing targeted prevention strategies for pancreatic cancer. To date, there seem to be limited publications related to the association between pancreatic steatosis and pancreas cancer.

Pancreatic steatosis is currently not a well-investigated issue [6,32,33,34,35]. The causes of pancreatic steatosis are also multiple in nature and are not well defined. There are also concerns regarding pancreatic steatosis causing a transient parenchymal change or a permanent change in the pancreas, as reported in an EUS study [13]. Pancreatic steatosis may be a reversible condition in pancreas tissue. A study has shown that weight loss resulting from metabolic surgery can reduce pancreatic fat composition [22]. However, pancreatic steatosis is also considered to be an overlooked factor for pancreatic cancer [15,21]. Pancreatic steatosis could be due to histological parenchymal changes, which may be related to acute or chronic pancreatitis [36]. However, our results demonstrate an important result showing that age, gender, diabetes, or surgery are not the most important factors surrounding pancreatic steatosis in pancreas cancer. However, chronic pancreatitis-related pancreatic steatosis seemed to be an important factor in pancreas cancer with pancreatic steatosis. A recent study also demonstrated a link between pancreatic steatosis and the risk of pancreatic cancer [16].

Accumulating evidence has suggested that pancreatic steatosis plays a role in pancreas cancer development in both animals and humans [15]. A retrospective study comparison of pancreatic cancer patients with or without pancreatic steatosis revealed that hypertension, congestive heart failure, and ischemic heart disease were the most important factors in pancreatic cancer with pancreatic steatosis [37]. Different from this study, chronic pancreatitis is the most significant factor in pancreatic steatosis for pancreas cancer patients. Additionally, a recent meta-analysis showed that pancreas cancer was found in 32% of pancreatic steatosis patients [25]. The results of one study regarding pancreatic steatosis in fatty liver and pancreatic disease showed that pancreatic steatosis occurred in 126 patients (44.8%) in patients with non-alcoholic fatty liver disease. Pancreatic steatosis patients seemed to be older (*p* = 0.0002) and more likely to have hypertension (*p* < 0.0001). The IPMN group had 18 patients (47.4%) with pancreatic steatosis, included more men than women (*p* = 0.0056), and was more likely to have patients diagnosed with hypertension (*p* = 0.0010) [16].

Metabolic dysfunction–associated steatotic liver disease (MASLD) is considered to be associated with extra-hepatic cancers [38]. The systemic effect of pancreas steatosis may be worthy of studying. It has been suggested that a fatty pancreas may contribute to the development of atherosclerosis in patients with MASLD [39]. A recent meta-analysis also disclosed the bi-directional association of hepatic and pancreatic association [40]. This study also confirms the strong association between MASLD and fatty pancreas. However, we tried to correlate pancreatic steatosis and hepatic steatosis. There was no significant association between pancreatic and hepatic steatosis in patients with pancreas malignancy in this study. Further complex correlation of gut microbiota, hepatic and pancreas steatosis through the gut–liver–pancreas axis is worth investigating [23].

The real mechanisms surrounding pancreatic steatosis are not yet well clarified. Most of the reviews and studies have indicated that chronic pancreatitis is one of the causes of pancreatic steatosis [6,7,8,41,42]. Our results showed that of the 30% of patients with pancreas cancer, pancreatic steatosis, and chronic pancreatitis were the most important factors. Targeting the causes of chronic pancreatitis and weight control could be considered a strategy for pancreas cancer prevention based on our findings. Due to alcohol also contributing to chronic pancreatitis, further studies on non-alcoholic and metabolic-related pancreatic steatosis may be worth investigating [21].

As this is a retrospective study, there are some limitations. First, the lipid profile seemed to not be complete in the study due to the enrollment of every newly diagnosed pancreas cancer patient in 2024, including patients whose pancreas cancer was accidentally discovered; thus, we did not have complete results regarding lipid profiles. Second, the lifestyles and habits of the patients (smoking or alcohol) were not complete in our study. The strength of this study is the diagnosis of pancreatic steatosis being based on either MRI or CT scan studies.

In conclusion, chronic pancreatitis was crucial in this study when diagnosing pancreas cancer with pancreatic steatosis. Due to the screening of pancreas cancer in high-risk patients being indicated, patients diagnosed with chronic pancreatitis and pancreas steatosis should consider these to be factors that would warrant a careful follow-up, as they may be at risk of pancreas cancer based upon this study’s results. Pancreatic steatosis also does not affect the prognosis of pancreas cancer. In addition, there was also no significant hepatic and pancreatic steatosis found among patients with pancreas cancer, despite obesity and BMI being a risk factor for pancreas cancer. In the future, investigating a larger number of patients and conducting a longitudinal follow-up for pancreas cancer development in individuals with chronic pancreatitis and pancreatic steatosis may be worthwhile.

## Figures and Tables

**Table 1:** Characteristics of pancreas cancer patients with or without fatty pancreas.

	Total (*n* = 101)		Fatty Pancreas				*p* Value
			No (*n* = 71)		Yes (*n* = 30)		
Age, mean ± SD	66.5	±11.6	65.3	±10.8	69.4	±13.1	0.112
Age, median (IQR)	66.0	(56.5–74)	65.0	(56–73)	70.5	(57–78.5)	0.112
BMI, mean ± SD	24.5	±14.3	24.8	±16.9	23.8	±3.6	0.274
BMI, median (IQR)	22.6	(20.6–25.7)	22.4	(20.6–25.4)	23.9	(21.2–26.4)	0.274
Sex, *n* (%)							0.617
Female	50	(49.5%)	34	(47.9%)	16	(53.3%)	
Male	51	(50.5%)	37	(52.1%)	14	(46.7%)	
DM, *n* (%)	30	(29.7%)	20	(28.2%)	10	(33.3%)	0.604
chronic pancreatitis, *n* (%)	26	(25.7%)	13	(18.3%)	13	(43.3%)	0.009 **
Staging, *n* (%)							0.345
I	19	(18.8%)	11	(15.5%)	8	(26.7%)	
II	13	(12.9%)	11	(15.5%)	2	(6.7%)	
III	16	(15.8%)	10	(14.1%)	6	(20.0%)	
IV	53	(52.5%)	39	(54.9%)	14	(46.7%)	
Pathology, *n* (%)	82	(81.2%)	57	(80.3%)	25	(83.3%)	0.720
OP, *n* (%)	26	(25.7%)	16	(22.5%)	10	(33.3%)	0.257
Death, *n* (%)	80	(79.2%)	59	(83.1%)	21	(70.0%)	0.138
Days, mean ± SD	231.1	±196.4	224.6	±190.4	246.5	±212.6	0.891
Days, median (IQR)	164.0	(66–372.5)	156.0	(68–337)	185.5	(22.3–439.8)	0.891

Mann–Whitney U test. Chi-Square test. ** *p* < 0.01.

**Table 2:** Factors associated with mortality in pancreas cancer.

	No (*n* = 21)		Yes (*n* = 80)		*p* Value
Age, mean ± SD	63.8	±11.7	67.3	±11.5	0.303
Age, median (IQR)	64.0	(55–73.5)	67.0	(60.3–74)	0.303
Sex, *n* (%)					0.846
Female	10	(47.6%)	40	(50.0%)	
Male	11	(52.4%)	40	(50.0%)	
BMI, mean ± SD	25.1	±3.0	24.4	±16.1	0.003 **
BMI, median (IQR)	25.4	(23.1–27.4)	22.2	(20.5–25)	0.003 **
Chol, mean ± SD	163.6	±69.0	145.8	±35.8	0.770
Chol, median (IQR)	139.5	(107.8–233.3)	144.5	(119.8–171.5)	0.770
TG, mean ± SD	164.9	±98.5	113.7	±56.2	0.066
TG, median (IQR)	136.0	(88–245)	102.0	(67–149)	0.066
LDL, mean ± SD	105.8	±60.9	83.7	±34.8	0.401
LDL, median (IQR)	94.0	(56.8–170)	79.5	(61.5–110.3)	0.401
Fatty pancreas, *n* (%)	9	(42.9%)	21	(26.3%)	0.138
DM, *n* (%)	6	(28.6%)	24	(30.0%)	0.899
Chronic pancreatitis, *n* (%)	7	(33.3%)	19	(23.8%)	0.371
Pathology, *n* (%)	21	(100%)	61	(76.3%)	0.011 *
OP, *n* (%)	14	(66.7%)	12	(15.0%)	<0.001 **
Staging, *n* (%)					<0.001 **
I	9	(42.9%)	10	(12.5%)	
II	4	(19.0%)	9	(11.3%)	
III	5	(23.8%)	11	(13.8%)	
IV	3	(14.3%)	50	(62.5%)	
Staging, *n* (%)					0.004 **
I	9	(42.9%)	10	(12.5%)	
II + III + IV	12	(57.1%)	70	(87.5%)	
Staging, *n* (%)					0.001 **
I + II	13	(61.9%)	19	(23.8%)	
III + IV	8	(38.1%)	61	(76.3%)	
Staging, *n* (%)					<0.001 **
I + II + III	18	(85.7%)	30	(37.5%)	
IV	3	(14.3%)	50	(62.5%)	
Fatty liver, *n* (%)	7	(33.3%)	6	(7.5%)	0.005 **

Mann-Whitney U test. Chi-Square test. Fisher’s Exact test. * *p* < 0.05, ** *p* < 0.01.

**Table 3:** Cox regression analysis for mortality in pancreas cancer patients.

	Univariate			Multivariate		
	HR	(95%CI)	*p* Value	HR	(95%CI)	*p* Value
Age	1.03	(1.01–1.05)	0.005 **	1.04	(1.02–1.07)	0.003 **
Sex						
Female	1.00					
Male	0.98	(0.63–1.52)	0.922			
BMI	1.00	(0.98–1.02)	0.885			
Chol	1.00	(0.99–1.00)	0.204			
TG	1.00	(0.99–1.00)	0.033 *	1.00	(0.99–1.00)	0.315
LDL	0.99	(0.98–1.00)	0.052			
Pancreatic steatosis	0.78	(0.47–1.28)	0.324			
DM	1.08	(0.67–1.74)	0.749			
Chronic pancreatitis	0.92	(0.55–1.54)	0.753			
Pathology	0.25	(0.14–0.44)	<0.001 **	0.66	(0.29–1.51)	0.325
OP	0.24	(0.13–0.45)	<0.001 **	0.36	(0.16–0.80)	0.013 *
Staging						
I	1.00			1.00		
II	1.40	(0.57–3.44)	0.466	1.38	(0.45–4.21)	0.577
III	1.37	(0.58–3.23)	0.471	0.91	(0.30–2.69)	0.857
IV	4.23	(2.11–8.46)	<0.001 **	2.75	(1.10–6.90)	0.031 *
Staging						
I	1.00					
II + III + IV	2.57	(1.32–5.00)	0.006 **			
Staging						
I + II	1.00					
III + IV	2.57	(1.53–4.33)	<0.001 **			
Staging						
I + II + III	1.00					
IV	3.44	(2.15–5.51)	<0.001 **			
Fatty liver	0.32	(0.14–0.75)	0.009 **	0.56	(0.22–1.41)	0.215

Cox regression. * *p* < 0.05, ** *p* < 0.01.

**Table 4:** Association of pancreatic steatosis and hepatic steatosis.

	Total (*n* = 101)		Pancreas Steatosis				*p* Value
			No (*n* = 71)		Yes (*n* = 30)		
Hepatic steatosis, *n* (%)							0.750
No	88	(87.1%)	61	(85.9%)	27	(90.0%)	
Yes	13	(12.9%)	10	(14.1%)	3	(10.0%)	

## Data Availability

The original contributions presented in the study are included in the article. Further inquiries can be directed to the corresponding author/s.
